# WWP2 regulates SIRT1‐STAT3 acetylation and phosphorylation involved in hypertensive angiopathy

**DOI:** 10.1111/jcmm.15538

**Published:** 2020-07-05

**Authors:** Ying Zhang, Shilong You, Yichen Tian, Saien Lu, Liu Cao, Yingxian Sun, Naijin Zhang

**Affiliations:** ^1^ Department of Cardiology The First Hospital of China Medical University Shenyang Liaoning China; ^2^ Key Laboratory of Medical Cell Biology Ministry of Education Institute of Translational Medicine China Medical University Liaoning Province Collaborative Innovation Center of Aging Related Disease Diagnosis and Treatment and Prevention Shenyang Liaoning China

**Keywords:** hypertensive angiopathy, post‐translational modification, SIRT1, STAT3, WWP2

## Abstract

WWP2 is a HECT‐type E3 ubiquitin ligase that regulates various physiological and pathological activities by binding to different substrates, but its function and regulatory mechanism in vascular smooth muscle cells (VSMCs) are still unknown. Here, we clarified the role of WWP2 in the regulation of SIRT1‐STAT3 and the impact of this regulatory process in VSMCs. We demonstrated that WWP2 expression was significantly increased in angiotensin II‐induced VSMCs model. Knockdown of WWP2 significantly inhibited angiotensin II‐induced VSMCs proliferation, migration and phenotypic transformation, whereas overexpression of WWP2 had opposite effects. In vivo experiments showed that vascular smooth muscle‐specific WWP2 knockout mice significantly relieved angiotensin II‐induced hypertensive angiopathy. Mechanistically, mass spectrometry and co‐immunoprecipitation assays identified that WWP2 is a novel interacting protein of SIRT1 and STAT3. Moreover, WWP2 formed a complex with SIRT1‐STAT3, inhibiting the interaction between SIRT1 and STAT3, then reducing the inhibitory effect of SIRT1 on STAT3, ensuing promoting STAT3‐K685 acetylation and STAT3‐Y705 phosphorylation in angiotensin II‐induced VSMCs and mice. In conclusion, WWP2 modulates hypertensive angiopathy by regulating SIRT1‐STAT3 and WWP2 suppression in VSMCs can alleviate hypertensive angiopathy vitro and vivo. These findings provide new insights into the treatment of hypertensive vascular diseases.

## 1 INTRODUCTION

Hypertension, characterized by elevated arterial blood pressure, is one of the major diseases that threaten human health. Vascular remodelling represents a pathological change in vascular structure due to hypertension, constituting one of the main causes of many hypertension‐associated complications such as atherosclerosis, stroke, kidney and heart failure.^1^, ^2^, ^3^ Vascular remodelling mainly includes vascular wall thickening and functional abnormalities of arterioles.

Vascular smooth muscle cells (VSMCs) are the main cellular components of vascular membranes. The proliferation and migration of VSMCs are critical cell‐based pathological changes in vascular diseases. Angiotensin II (AngII) binds to VSMCs’ AngII type 1 receptor to activate STAT3 in order to initiate the proliferation and migration of VSMCs, representing key effects in vascular lesions caused by hypertension.^4^, ^5^, ^6^ In addition, VSMCs include contractile and synthetic phenotypes according to structural and functional differences. The main function of contractile VSMCs is to contract and maintain the vascular wall tension, whereas synthetic VSMCs contain large amounts of organelles and a small number of muscle fibres, which can promote VSMCs proliferation and migration. VSMCs can be transformed from contractile to synthetic types under the action of AngII‐induced STAT3 activation, which is the basis of VSMCs involvement in angiopathy caused by hypertension.^7^, ^8^, ^9^

STAT3 activation mainly includes acetylation and phosphorylation of STAT3, which is regulated by deacetylase SIRT1.^10^, ^11^, ^12^, ^13^, ^14^, ^15^ SIRT1 deacetylates STAT3, ensuing inhibiting STAT3 phosphorylation, thus exerting its role in inhibiting AngII‐induced VSMCs proliferation, migration and phenotypic transformation.^10^, ^11^, ^12^, ^13^, ^14^, ^15^ Therefore, SIRT1‐dependent STAT3 acetylation and phosphorylation are critical to VSMCs proliferation, migration and phenotypic transformation during hypertension condition. However, the regulatory mechanism among these events remains incompletely understood.

The E3 ubiquitin ligase WWP2 controls many biological processes such as the cell cycle, cell division, immune response, antigen presentation, apoptosis and cell signalling.^16^, ^17^, ^18^, ^19^ WWP2 not only degrades substrates, but also regulates genes post‐transcriptionally.^20^ Studies have demonstrated that WWP2 dysfunction can cause many diseases, including maxillofacial deformity, skeleton damage and embryonic dysplasia.^16^, ^21^, ^22^ However, no study has assessed whether WWP2 is involved in the physiological and pathological processes of VSMCs. Therefore, investigating the role and molecular mechanism of WWP2 in VSMCs phenotypic transformation as well as proliferation and migration may provide new insights into the treatment of hypertensive vascular diseases such as stroke, kidney and heart failure.

In this study, it was found for the first time that the expression of WWP2 is elevated in AngII‐induced VSMCs proliferation, migration and phenotypic transformation model. In addition, WWP2 knockdown in VSMCs significantly relieved AngII‐induced VSMCs proliferation, migration and phenotypic transformation, whereas WWP2 overexpression promoted this phenomenon. In vascular smooth muscle‐specific WWP2 knockout mice, significantly relieved in AngII‐induced hypertensive angiopathy was observed. Therefore, In vivo and in vitro vascular smooth muscle‐specific WWP2 knockout significantly alleviated hypertensive angiopathy. Mechanistically, mass spectrometry and co‐immunoprecipitation assays identified that STAT3 and SIRT1 were two novel proteins interacting with WWP2. Furthermore, WWP2 formed a complex with STAT3‐SIRT1, competing for the interaction between SIRT1 and STAT3, then reducing the inhibitory effect of SIRT1 on STAT3, ensuing enhancing STAT3‐K685 acetylation and STAT3‐Y705 phosphorylation, which finally promoted AngII‐induced proliferation, migration and phenotypic transformation in VSMCs and hypertensive angiopathy in mice.

## 2 MATERIALS AND METHODS

### 2.1 Vascular smooth muscle‐specific WWP2 knockout mice experiments

Conditional vascular smooth muscle WWP2 knockout (*SM22α Cre+;WWP2^FL/FL^*) mice and the control (*SM22α Cre‐;WWP2^FL/FL^*) mice were established by Shanghai Biomodel Organism Science & Technology Development. Conditional vascular smooth muscle WWP2 knockout in vivo was assessed by immunofluorescence and Western blot for aortic vascular tissue of *SM22α Cre+;WWP2^FL/FL^* and *SM22α Cre‐;WWP2^FL/FL^* mice. Conditional vascular smooth muscle WWP2 knockout mice have been shown to be effective (Figure 6A‐C). Specific pathogen‐free (SPF) male mice (8‐10 weeks) were assessed. Hypertensive angiopathy model was performed with NaCl and AngII (A9525, Sigma, USA; 1.5 mg kg^−1^ d^−1^), respectively, for 2 weeks with Alzet minipumps (Alzet, model 2002; 0.5 μL/h).^23^, ^24^ *SM22α Cre+; WWP2^FL/FL^* and *SM22α Cre‐; WWP2^FL/FL^* mice (N = 9/group) were randomized prior to anaesthesia via inhalation of isoflurane/oxygen (2%, ~1500 mL/min; depth monitored via lack of paw withdrawal reflex) and then implanted with osmotic minipumps (Alzet) subcutaneously in the back of mice. Blood pressure measurement was carried out daily by the tail‐cuff method. Cervical dislocation after isoflurane inhalation anaesthesia was used for mice euthanasia. All animals handling complied with animal welfare regulations of China Medical University. The Animal Subjects Committee of China Medical University approved the animal study protocol. The investigation conforms to the guide for the care and use of laboratory animals published by the US National Institutes of Health (NIH Publications No. 8023, revised 1978).

### 2.2 Micro‐CT scanning and 3D reconstruction

Micro‐computed tomography (micro‐CT) on a micro‐CT‐Imaging SkyScan 1276 (Bruker, Germany) was carried out at 70 kV (200 µA), with 1237 projections (1520 × 1264) acquired within 6 minutes 43 seconds under continuous tube rotation. AngII signals were acquired in 20 × 20 × 20‐μm^3^ voxels with DataViewer (Bruker, Germany), with ring artefact correction. Then, the images were reconstructed and data visualized with NRecon (Bruker); CTAn (Bruker) was used for further evaluation. After 3D spine segmentation by interactively delineating the aorta in 100 and 200 slices (2 and 4 mm, respectively; Figure 6D,E), artery and vein circumferences were evaluated, with average cardiac tissue brightness after contrast agent injection into an artery and pre‐contrast agent administration set at 100 and 0%, respectively.^25^ The slices of each mouse were taken from the same position.

### 2.3 Immunohistochemical (IHC) analysis

Aortic vascular tissue specimens from mice were fixed with 4% formalin (4 hours), paraffin embedded and sectioned at 5‐µm, and the aortic slices of each mouse were taken from the same position. After xylene dewaxing and rehydration by graded ethyl alcohol, the sections underwent haematoxylin and eosin (H&E) staining.

### 2.4 Cell culture, cell transfections and co‐immunoprecipitation

Human aortic vascular smooth muscle cells (HAVSMCs) were provided by Cambrex (China Center for Type Culture Collection, China) and maintained in H‐Dulbecco's modified Eagle medium (H‐DMEM) (HyClone, USA) containing 10% foetal bovine serum (HyClone) in a humid environment with 5% CO_2_ at 37°C. HAVSMCs were passaged 4‐6 times before use. Transfection was performed with Lipofectamine 3000 (Invitrogen, USA) as directed by the manufacturer (plasmid/transfection reagent = 1 µg/2.4 µL). For co‐immunoprecipitation, cells underwent two washes and lysis with flag lysis buffer (50 mmol/L Tris, 137 mmol/L NaCl, 1 mmol/L EDTA, 10 mmol/L NaF, 0.1 mmol/L Na3VO4, 1% NP‐40, 1 mmol/L DTT, and 10% glycerol, pH 7.8) with freshly prepared protease inhibitors. Cell lysates underwent incubation with antibodies (antibody/cell lysates = 1 µg/mg; 3 hours) and 30 µL of protein A/G immunoprecipitation magnetic beads (B23202; Biotool, USA) (12 hours, 4°C). The resulting immuno‐complexes were assessed by SDS‐PAGE.

### 2.5 HAVSMCs WWP2 knockdown methods and Plasmid construction

Control siRNAs and WWP2 siRNAs were provided by RIBOBIO (China). WWP2 knockdown was performed with the jetPRIME transfection reagent (PolyPlus, France) (siRNA/transfection reagent = 12.5pmoles/µL). Three target sequences (WWP2 siRNA‐1: GATCTGGGAAATGTGCCTA; WWP2 siRNA‐2: GGTGCTTCAGCCAGAACAA; WWP2 siRNA‐3: CGGACGTGTCTATTATGTT) were assessed for excluding off‐target effects. WWP2 knockdown efficiency was confirmed by immunoblot.

Plasmids encoding the full‐length human WWP2 (Shanghai Genechem) were cloned to HA‐tagged destination vectors according to immunoprecipitation and immunoblotting needs.

### 2.6 Antibodies and reagents

Antibodies to polyclone rabbit anti‐WWP2 (1:1000; Abcam, USA; 1:500; Proteintech, USA), monoclonal rabbit anti‐HA (1:1000; Cell Signaling Technology, USA), polyclone rabbit anti‐STAT3‐K685AC (1:1000; Cell Signaling Technology, USA), polyclone rabbit anti‐STAT3‐Y705P (1:1000; Cell Signaling Technology, USA), polyclonal rabbit anti‐STAT3 (1:1000; Proteintech, USA), polyclonal rabbit anti‐SIRT1 (1:1000; Proteintech, USA), polyclonal rabbit anti‐SM22α (1:1000; Proteintech, USA), polyclonal rabbit anti‐α‐SM‐actin (1:1000; Proteintech, USA), monoclonal rabbit anti‐MMP9 (1:200; Santa, USA), polyclonal rabbit anti‐MMP2 (1:500; Proteintech, USA), polyclonal rabbit anti‐PCNA (1:1000; Proteintech, USA), polyclonal rabbit anti‐β‐Tubulin (1:2000; Proteintech, USA) and monoclonal mouse anti‐GAPDH (1:2000; Abcam, USA) were purchased commercially. Protein A/G immunoprecipitation magnetic beads were obtained from Biotool and used for immunoprecipitation.

### 2.7 Cell viability, migration and phalloidin staining assay

Cell Counting Kit‐8 (CCK‐8; Biotool, USA) was employed to assess cell viability. HAVSMCs were seeded into a 96‐well plate at 3x10^3^ cells/well in H‐DMEM containing 10% FBS and underwent transfection with control and HA‐WWP2 plasmids using Lipofectamine 3000 (Invitrogen, California, USA) (plasmid/transfection reagent = 1 µg/2.4 µL), respectively, and control siRNAs and WWP2 siRNAs using jetPRIME (PolyPlus, France) (siRNA/transfection reagent = 12.5pmoles/µL), respectively. AngII treatment was used for 24 hours after starvation in serum‐free medium for 24 hours, and 100 µL of CCK‐8 reagent was added per well for 2 hours. Absorbance was determined at 450 nm on a Bio‐Rad microplate reader (Model 680; Bio‐Rad, USA).

Cell migration was assessed in transwell plates (Corning Life Sciences, USA). A total of 5 × 10^3^ cells were seeded into the upper chambers of 24‐well plates. AngII treatment was used for 24 hours after starvation in serum‐free medium for 24 hours. The top chambers were supplemented with serum‐free H‐DMEM, whereas the lower ones contained the complete medium with 10% FBS. Upon culture at 37°C overnight, cotton swabs were employed to remove non‐migrating cells; those that migrated underwent fixation with chilled methanol and staining with crystal violet, and were counted in totally 5 random fields.

AngII treatment was used for 24 hours after starvation in serum‐free medium for 24 hours, and Phalloidin (Molecular Probes, USA) staining of HAVSMCs was performed after fixation with 4% formalin (20 minutes) and permeabilization with 0.5% Triton X‐100 (30 minutes), as directed by the manufacturer. Cell morphology and actin filaments were observed under a fluorescence microscope (Olympus).

### 2.8 Western blot

Upon treatment, cell lysis was performed with co‐immunoprecipitation buffer. The lysates were submitted to centrifugation (13 300 rpm; 20 minutes, 4°C) for protein extraction. The BCA protein assay kit (Dingguo Changsheng Biotechnology, China) was employed for total protein quantitation. Equal amounts of protein (40 μg) were resolved by SDS = PAGE and electro‐transferred onto PVDF membranes. Then, 5% bovine serum albumin (BSA) in Tris‐buffered saline‐Tween (TBST) was employed for blocking in ambient conditions (1 hour), followed by incubation overnight (4°C) with primary antibodies. GAPDH or β‐Tubulin was used as a loading control. Image J v1.46 (National Institutes of Health, USA) was employed for analysis.

### 2.9 Statistical analysis

Data are mean ± standard deviation (SD). Homogeneity of variance was evaluated by the F test (group pair) or Brown‐Forsythe test (multiple groups). The Shapiro‐Wilk test was performed for assessing data normality. Student's *t* test and Welch t test were employed to assess data of group pairs with normal and skewed distributions, respectively (two groups). ANOVA and indicated non‐parametric tests were performed to compare multiple groups. One‐way ANOVA and two‐way ANOVA were performed for comparing groups for single and two factors, respectively, and followed by Bonferroni *post hoc* testing. *P* values were adjusted for multiple comparisons when applicable. All data were analysed by SPSS 22.0 (SPSS, USA), and *P* < 0.05 was considered statistically significant.

## 3 RESULT

### 3.1 WWP2 interacts with STAT3 and promotes STAT3 acetylation and phosphorylation

**FIGURE 1:**
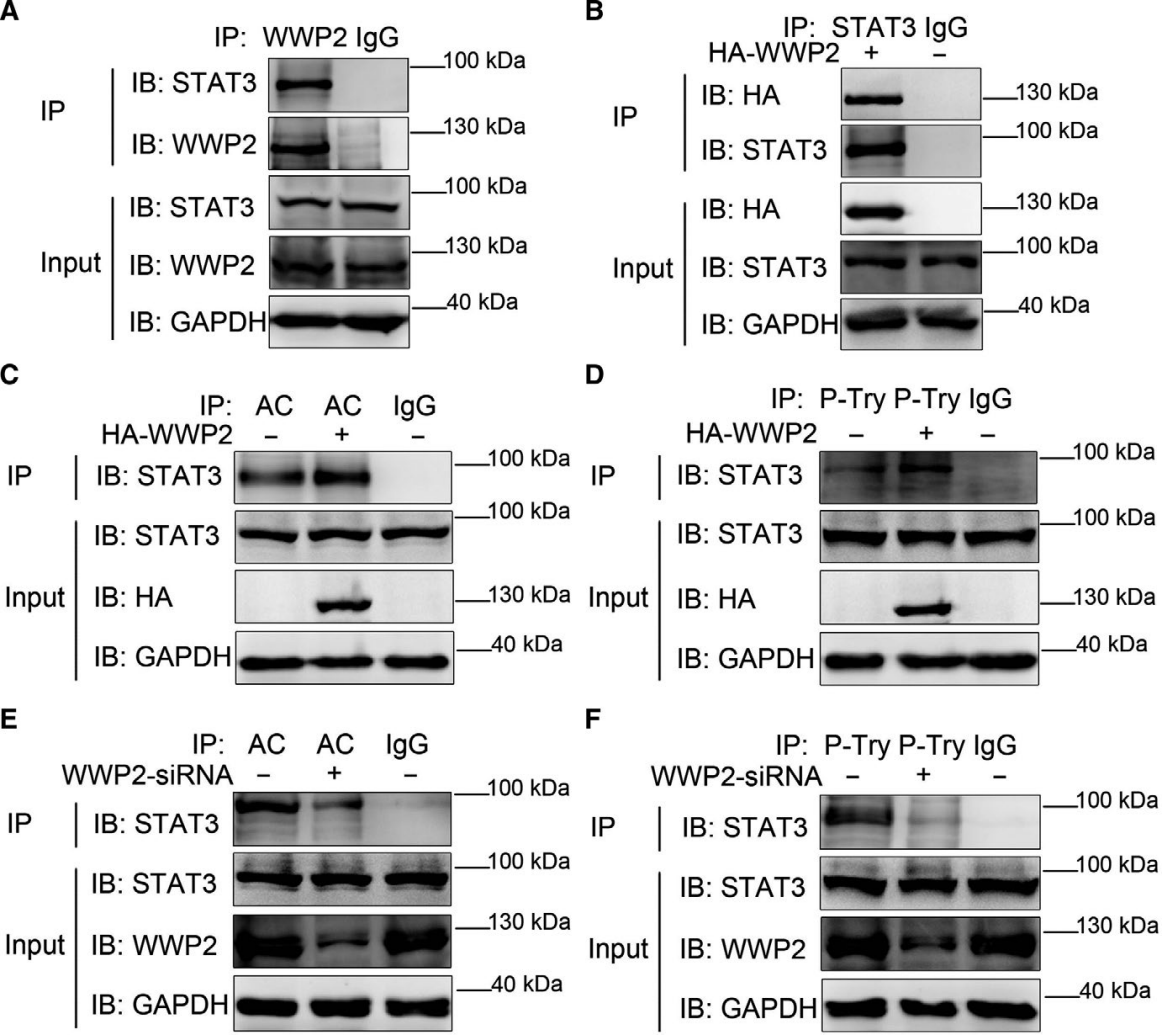
WWP2 interacts with STAT3 and promotes STAT3 acetylation and phosphorylation. A, In HAVSMCs, endogenous co‐immunoprecipitation was performed to assess the interaction between WWP2 and STAT3. B, As did exogenous HA‐WWP2 and STAT3. C, HA‐WWP2 was transfected into HAVSMCs. Pan‐acetylation was isolated by co‐immunoprecipitation, and STAT3 acetylation levels were assessed with anti‐STAT3 antibody. D, HA‐WWP2 was transfected into HAVSMCs. P‐Tyr was isolated by co‐immunoprecipitation, and STAT3 phosphorylation levels were assessed with anti‐STAT3 antibody. E, WWP2 siRNA was transfected into HAVSMCs. Pan‐acetylation was isolated by co‐immunoprecipitation, and STAT3 acetylation levels were assessed with anti‐STAT3 antibody. F, WWP2 siRNA was transfected into HAVSMCs. P‐Tyr was isolated by co‐immunoprecipitation, and STAT3 phosphorylation levels were assessed with anti‐STAT3 antibody

STAT3 is an important regulator in VSMCs, and its acetylation and phosphorylation are critical to VSMCs proliferation, migration and phenotypic transformation.^4^, ^5^, ^6^ Mass spectrometry and co‐immunoprecipitation assays identified that the E3 ubiquitin ligase WWP2 was a novel protein interacting with STAT3. WWP2 was found to interact with STAT3 by internal and external co‐immunoprecipitation assays (Figure 1A,B).

As WWP2 was an E3 ubiquitin ligase, its effects on STAT3 expression were firstly detected, and the results showed that WWP2 did not regulate STAT3 expression (Supplementary Figure S1A,B). However, co‐immunoprecipitation assays results showed that WWP2 promoted the acetylation and phosphorylation levels of STAT3 (Figure 1C,D), whereas its knockdown reduced the acetylation and phosphorylation levels of STAT3 (Figure 1E,F).

The above results confirmed that WWP2, as a new interaction substrate of STAT3, promoted the acetylation and phosphorylation levels of STAT3, which may be the key for regulating VSMCs proliferation, migration and phenotypic transformation.

### 3.2 WWP2 forms a complex with SIRT1‐STAT3, competing for the interaction between SIRT1 and STAT3

**FIGURE 2:**
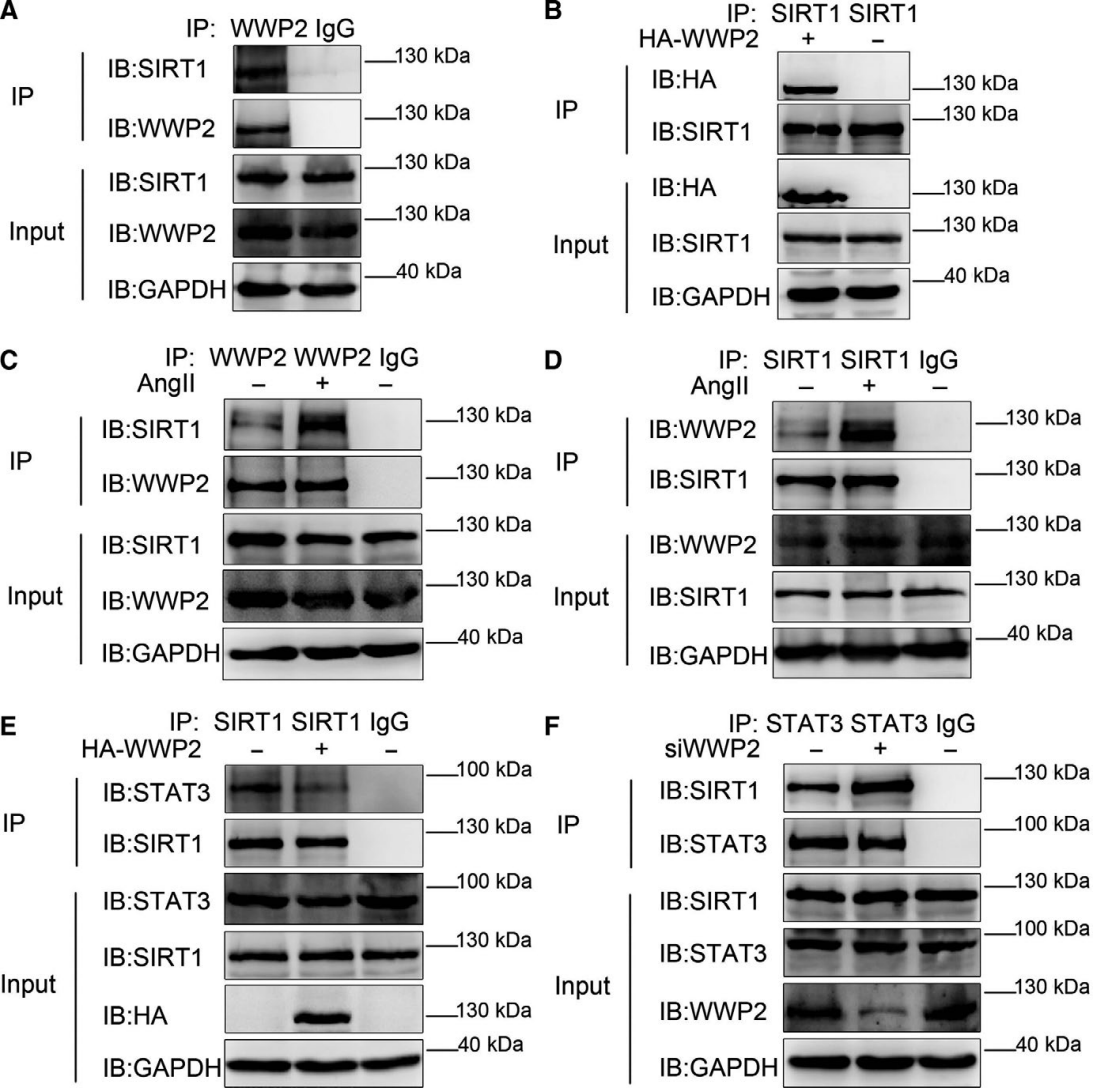
WWP2 forms a complex with SIRT1‐STAT3, competing for the interaction between SIRT1 and STAT3. A and B, In HAVSMCs, endogenous co‐immunoprecipitation was performed to assess the interaction between WWP2 and SIRT1. B, As did exogenous HA‐WWP2 and SIRT1. C and D, Endogenous co‐immunoprecipitation was performed to assess the interaction between WWP2 and SIRT1 with the addition of 10^−6^ mol/L angiotensin II. E, HA‐WWP2 was transfected into HAVSMCs. Endogenous co‐immunoprecipitation between SIRT1 and STAT3 was assessed. F, WWP2 siRNA was transfected into HAVSMCs. Endogenous co‐immunoprecipitation between SIRT1 and STAT3 was assessed

As WWP2 is neither acetylase nor phosphokinase, we next explored the mechanism by which WWP2 promotes the acetylation and phosphorylation levels of STAT3. Mass spectrometry and co‐immunoprecipitation assays results showed that SIRT1 was identified as another new physiological substrate of WWP2. Internal and external co‐immunoprecipitation assays confirmed that WWP2 interacted with SIRT1 (Figure 2A,B). Moreover, the interaction between WWP2 and SIRT1 was enhanced after stimulation by AngII in VSMCs (Figure 2C,D).

SIRT1 is an important inhibitory factor of AngII‐induced VSMCs phenotypic transformation as well as proliferation and migration, acting by decreasing STAT3 acetylation and phosphorylation.^10^, ^11^, ^12^, ^13^, ^14^, ^15^ Therefore, we further explored whether WWP2 promotes STAT3 acetylation and phosphorylation by inhibiting the effect of SIRT1 on STAT3. As expected, co‐immunoprecipitation assays results showed that WWP2 overexpression decreased the interaction between SIRT1 and STAT3 (Figure 2E), whereas WWP2 knockdown increased the interaction between SIRT1 and STAT3 (Figure 2F).

The above results revealed that WWP2 competed with the interaction between SIRT1 and STAT3, ensuing reducing SIRT1‐regulated deacetylation and dephosphorylation of STAT3, which promotes the acetylation and phosphorylation levels of STAT3.

### 3.3 WWP2 antagonizes SIRT1‐inhibited STAT3‐K685 acetylation and STAT3‐Y705 phosphorylation in AngII‐induced VSMCs

**FIGURE 3:**
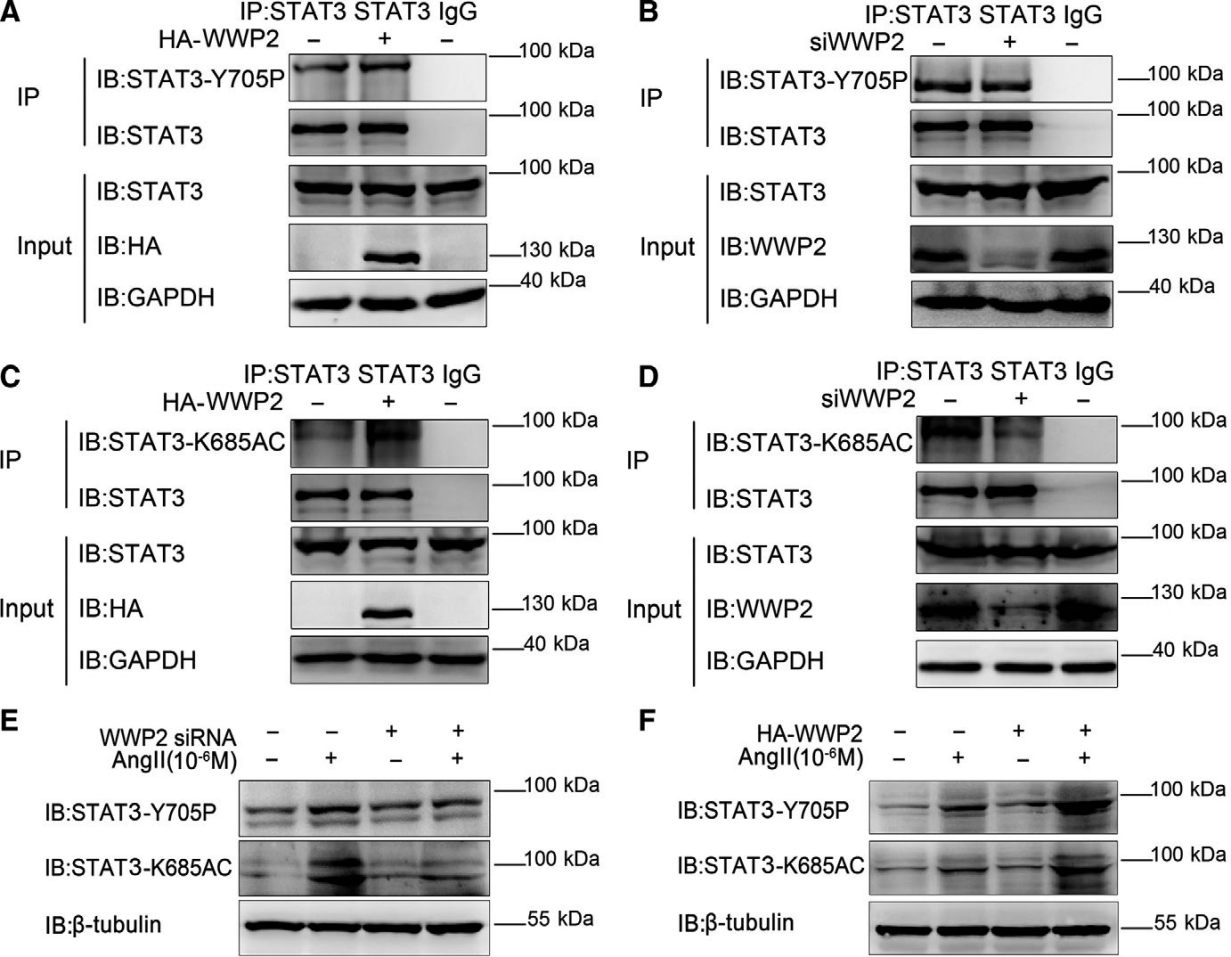
WWP2 promotes levels of STAT3‐K685 acetylation and STAT3‐Y705 phosphorylation. A and B, HA‐WWP2 and WWP2 siRNA were transfected into HAVSMCs. STAT3 was isolated by co‐immunoprecipitation, and STAT3‐Y705 phosphorylation levels were assessed with anti‐STAT3‐Y705 antibody. C and D, HA‐WWP2 and WWP2 siRNA were transfected into HAVSMCs. STAT3 was isolated by co‐immunoprecipitation, and STAT3‐K685 acetylation levels were assessed with anti‐STAT3‐K685 antibody. E and F, HA‐WWP2 and WWP2 siRNA were transfected into HAVSMCs with or without treatment of 10^−6^ mol/L AngII after serum‐free medium starvation. STAT3‐K685 acetylation and STAT3‐Y705 phosphorylation levels were assessed with anti‐STAT3‐K685 and anti‐STAT3‐Y705 antibodies

Previous studies have shown that SIRT1 inhibits STAT3 acetylation and phosphorylation mainly acting on STAT3‐K685 and STAT3‐Y705, respectively.^15^ Therefore, in order to provide the most direct and important evidence that WWP2 promotes STAT3 acetylation and phosphorylation by inhibiting the effect of SIRT1 on STAT3, we further clarify the loci in which WWP2 promotes STAT3 acetylation and phosphorylation. In this study, STAT3 acetylation and phosphorylation locus antibodies were used for co‐immunoprecipitation assays after WWP2 overexpression and knockdown in VSMCs, respectively. The results showed that WWP2 overexpression increased STAT3‐K685 acetylation (Figure 3C) and STAT3‐Y705 phosphorylation (Figure 3A). Meanwhile, knockdown of WWP2 reduced STAT3‐K685 acetylation (Figure 3D) and STAT3‐Y705 phosphorylation (Figure 3B).

In addition, we validated this mechanism in AngII‐induced VSMCs model. The results showed that overexpression of WWP2 increased AngII‐induced STAT3‐K685 acetylation and STAT3‐Y705 phosphorylation in VSMCs (Figure 3F). Conversely, knockdown of WWP2 decreased AngII‐induced STAT3‐K685 acetylation and STAT3‐Y705 phosphorylation in VSMCs (Figure 3E).

Taken together, WWP2 formed a complex with SIRT1‐STAT3, inhibiting the interaction between SIRT1 and STAT3, then reducing the inhibitory effect of SIRT1 on STAT3, ensuing promoting STAT3‐K685 acetylation and STAT3‐Y705 phosphorylation in AngII‐induced VSMCs.

### 3.4 WWP2 promotes AngII‐induced VSMCs proliferation and migration, and up‐regulates PCNA, MMP2 and MMP9 expression

STAT3 acetylation and phosphorylation are critical to VSMCs proliferation, migration and phenotypic transformation.^4^, ^5^, ^6^ Therefore, the effects of WWP2 on VSMCs proliferation, migration and phenotypic transformation were further assessed after identifying the specific mechanism of WWP2 in regulating the SIRT1‐STAT3 complex. The results showed that AngII significantly induced VSMCs proliferation and migration as well as phenotypic transformation (Supplementary Figure S2), and WWP2 expression gradually increased with AngII concentration (Supplementary Figure S3).

**FIGURE 4:**
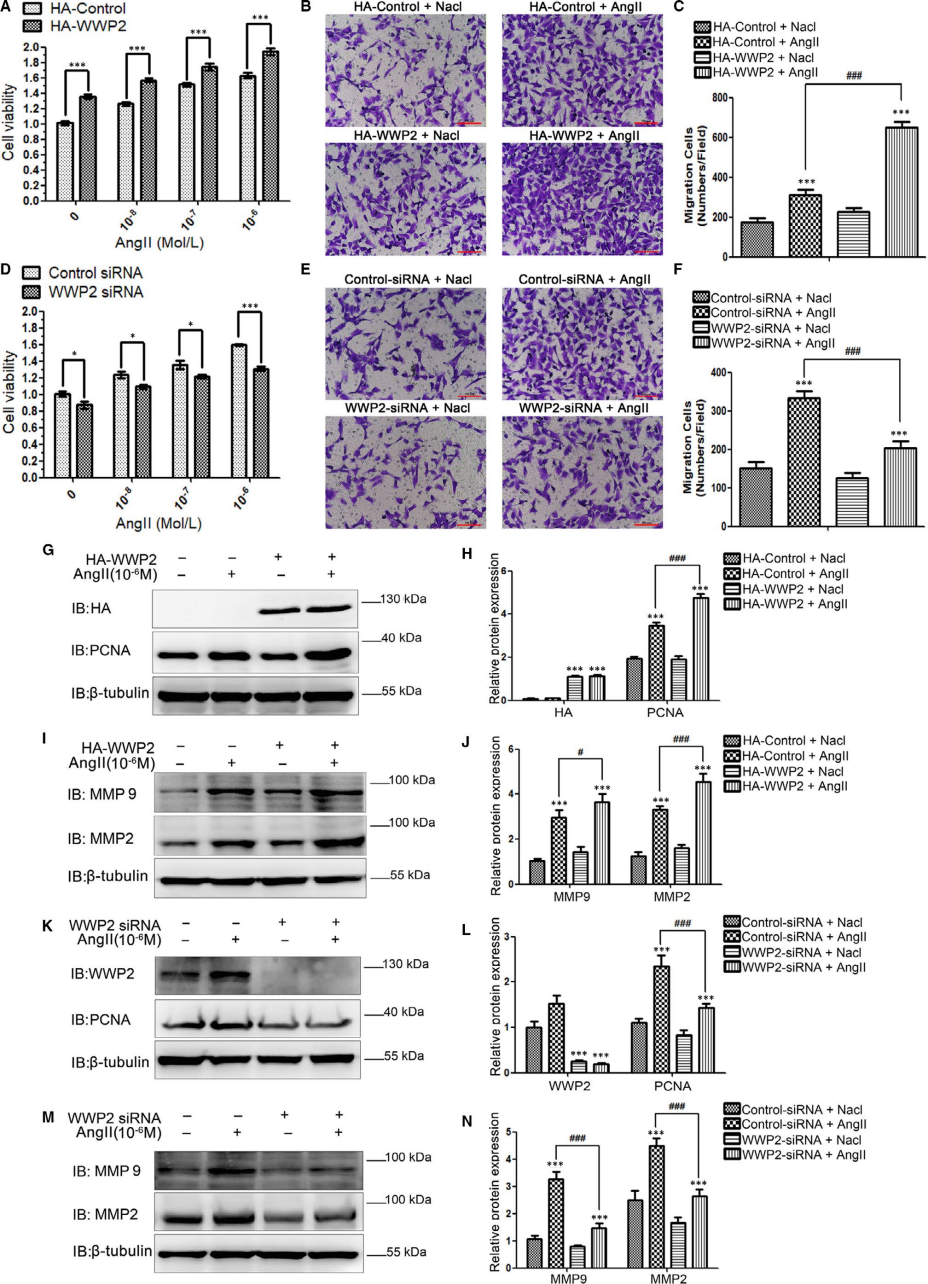
WWP2 promotes AngII‐induced HAVSMCs proliferation and migration, and up‐regulates the expression of PCNA, MMP2 and MMP9. A, HAVSMCs were transfected with the HA‐control or HA‐WWP2 plasmid for 36 h and treated with 0, 10^−8^, 10^−7^ and 10^−6^ mol/L AngII for 24 h after serum‐free medium starvation, respectively. CCK8 was used to assess HAVSMCs viability, and quantitated data were shown as means ± SD (***P < .001, unpaired Student's *t* test). D, As did HAVSMCs were transfected with the control siRNA or WWP2 siRNA. B and C, HAVSMCs were transfected with the HA‐control or HA‐WWP2 plasmid for 36 h and treated with or without 10^−6^ mol/L AngII for 24 h after serum‐free medium starvation. Transwell was used to assess HAVSMCs migration, and quantitated data were shown as means ± SD (***P < .001, unpaired Student's *t* test; ^###^ *P* < .001, two‐way ANOVA with Bonferroni *post hoc* testing). Scale bar 100 µm. E and F, As did HAVSMCs were transfected with the control siRNA or WWP2 siRNA. G‐J, HAVSMCs were transfected with the HA‐control or HA‐WWP2 plasmid for 36 h and treated with or without 10^−6^ mol/L AngII for 24 h after serum‐free medium starvation. PCNA, MMP2 and MMP9 were detected by Western blot, and quantitated data were shown as means ± SD (***P < .001, unpaired Student's *t* test; ^#^ *P* < .05, ^###^ *P* < .001, two‐way ANOVA with Bonferroni *post hoc* testing). K‐N, As did HAVSMCs were transfected with the control siRNA or WWP2 siRNA. All the experiments were repeated three times

In addition, CCK8 and transwell experiment confirmed that overexpression of WWP2 significantly increased AngII‐induced VSMCs proliferation (Figure 4A) and migration (Figure 4B,C), whereas its knockdown inhibited AngII‐induced VSMCs proliferation (Figure 4D) and migration (Figure 4E,F). Furthermore, overexpression of WWP2 significantly increased AngII‐induced the expression of proliferation and migration protein makers PCNA (Figure 4G,H), MMP2 and MMP9 (Figure 4I,J) in VSMCs, whereas its knockdown had opposite effects (Figure 4K‐N).

The above results showed that WWP2 promoted AngII‐induced VSMCs proliferation and migration, and its knockdown in VSMCs might become an important tool for suppressing hypertensive VSMCs proliferation and migration.

### 3.5 WWP2 promotes AngII‐induced VSMCs phenotypic transformation and down‐regulates the contractile phenotypic proteins α‐SM‐actin and SM22α

**FIGURE 5:**
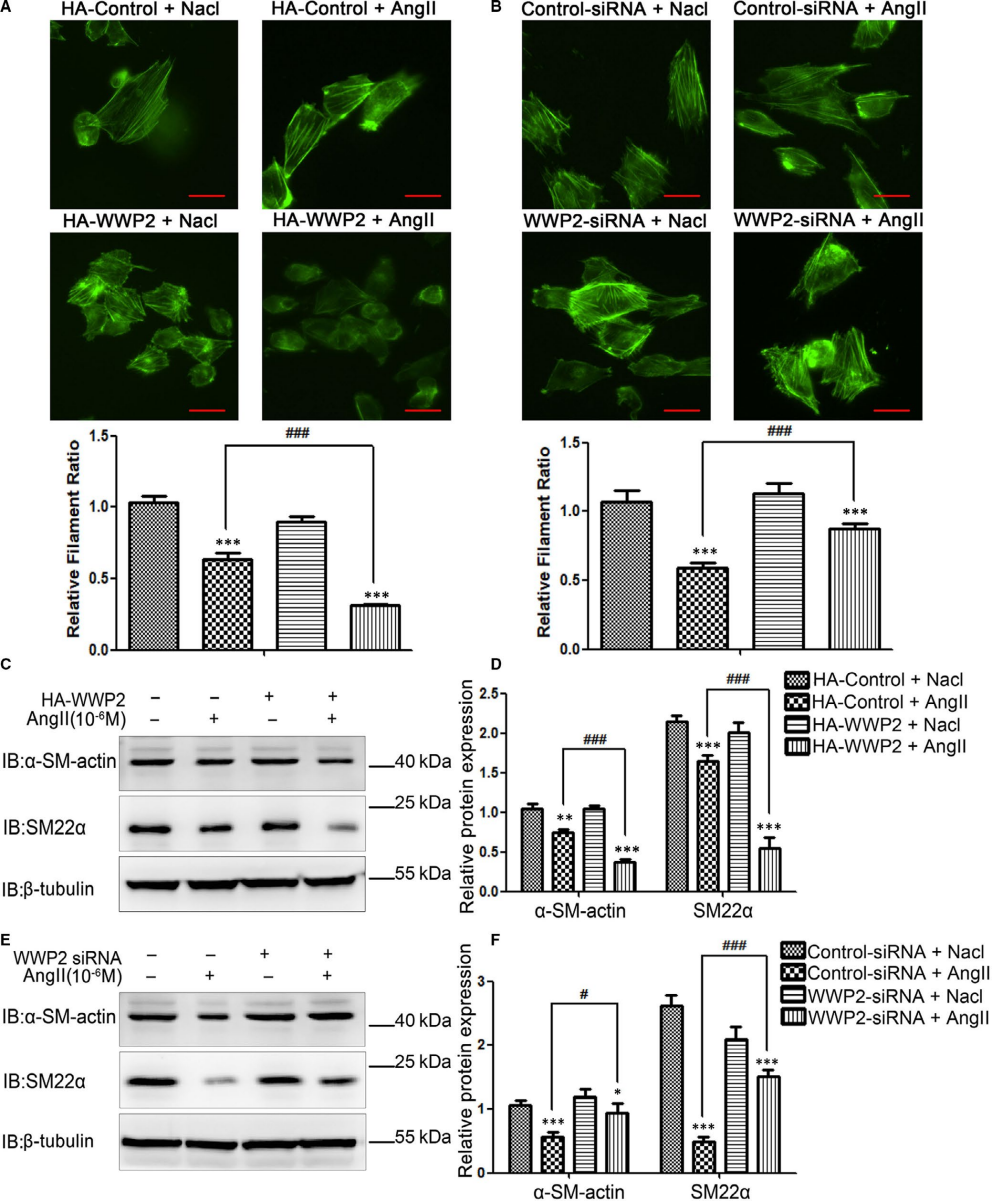
WWP2 promotes AngII‐induced HAVSMCs phenotypic transformation and decreases the expression of α‐SM‐actin and SM22α proteins. A, HAVSMCs were transfected with the HA‐control or HA‐WWP2 plasmid for 36 h and treated with or without 10^−6^ mol/L AngII for 24 h after serum‐free medium starvation. Phalloidine dye was used to assess HAVSMCs phenotypic transformation and intracellular myofilaments were labelled with green fluorescence. Quantitated data were shown as means ± SD (***P < .001, unpaired Student's *t* test; ^###^ *P* < .001, two‐way ANOVA with Bonferroni *post hoc* testing). Scale bar 50 µm. B, As did HAVSMCs were transfected with the control siRNA or WWP2 siRNA. C and D, HAVSMCs were transfected with the HA‐control or HA‐WWP2 plasmid for 36 h and treated with or without 10^−6^ mol/L AngII for 24 h after serum‐free medium starvation. α‐SM‐actin and SM22α were detected by Western blot, and quantitated data were shown as means ± SD (**P < .01, ***P < .001, unpaired Student's t test; ^###^ *P* < .001, two‐way ANOVA with Bonferroni *post hoc* testing). E and F, As did HAVSMCs were transfected with the control siRNA or WWP2 siRNA. All the experiments were repeated three times

Next, this study assessed the effects of WWP2 on AngII‐induced VSMCs phenotypic transformation. The results showed that WWP2 overexpression significantly increased AngII‐induced VSMCs phenotypic transformation (Figure 5A), whereas its knockdown inhibited AngII‐induced VSMCs phenotypic transformation (Figure 5B). Additionally, overexpression of WWP2 significantly decreased AngII‐induced expression of contractile phenotype proteins α‐SM‐actin and SM22α (Figure 5C,D) in VSMCs, whereas WWP2 knockdown had opposite effects (Figure 5E,F).

Taken altogether, these findings indicated that WWP2 formed a complex with SIRT1‐STAT3, inhibiting the interaction between SIRT1 and STAT3, then reducing the inhibitory effect of SIRT1 on STAT3, ensuing promoting STAT3‐K685 acetylation and STAT3‐Y705 phosphorylation, which finally promoted AngII‐induced VSMCs proliferation and migration as well as phenotypic transformation, and WWP2 silencing in VSMCs may become a critical tool in controlling vascular diseases associated with AngII‐induced VSMCs proliferation, migration and phenotypic transformation, such as hypertensive angiopathy‐hypertensive vascular thickening.

### 3.6 Mice vascular smooth muscle‐specific WWP2 knockout reduces STAT3‐K685 acetylation and STAT3‐Y705 phosphorylation, and relieves hypertensive arteries and veins angiopathy

**FIGURE 6:**
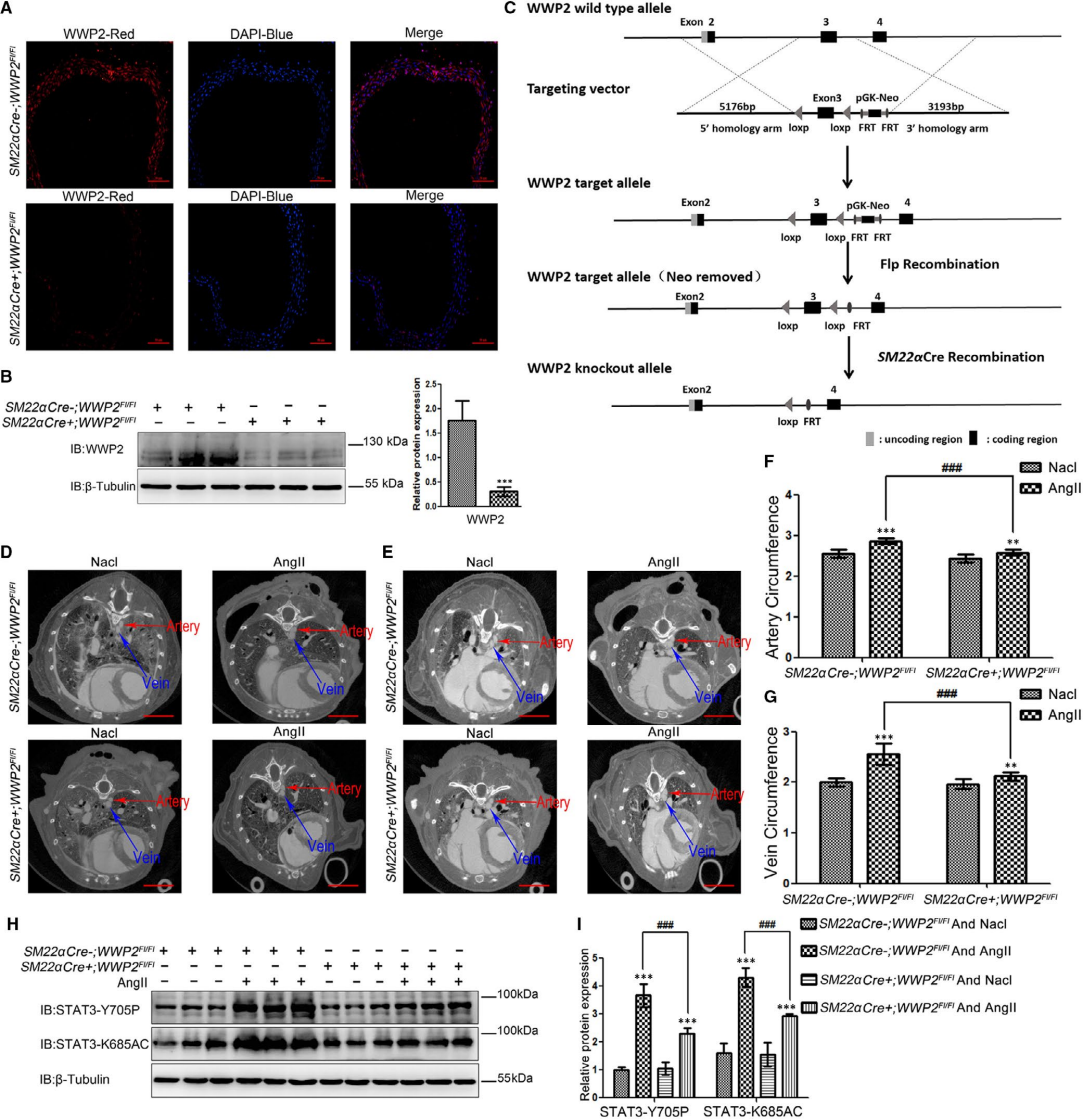
Mice vascular smooth muscle‐specific WWP2 knockout reduces STAT3‐K685 acetylation and STAT3‐Y705 phosphorylation, and relieves hypertensive arteries and veins angiopathy. A, Immunofluorescence of blood vessel tissues was performed to assess WWP2 expression levels in *SM22α Cre‐;WWP2^FL/FL^* and *SM22α Cre‐;WWP2^FL/FL^* mice. Scale bar 50 µm. B, Total protein was obtained from blood vessel tissues of *SM22α Cre‐;WWP2^FL/FL^* and *SM22α Cre‐;WWP2^FL/FL^* mice. Western blot analyses were performed to assess WWP2 expression levels, and quantitated data were shown as means ± SD (each group of mice, n = 9; ***P < .001, unpaired Student's *t* test). C, Construction strategy of gene conditional elimination targeting vector. D and E, Aortic blood vessel detection by micro‐CT after administration of blood pool contrast solution containing iodine (eXIA 160XL), which allows a spatial resolution of 20 μm voxels 2D cross‐sectional images in *SM22α Cre‐;WWP2^FL/FL^* and *SM22α Cre‐;WWP2^FL/FL^* mice delineating from the heart pre‐contrast agent injection (0%) to the aorta boundaries in 100 slices and 200 slices. Scale bar 4 mm. F and G, Vein circumference and artery circumference were determined by in vivo micro‐CT, and the slices of each mouse were taken from the same position. Quantitated data were shown as means ± SD (each group of mice, n = 9; **P < .01, ***P < .001, unpaired Student's *t* test; ^###^ *P* < .001, two‐way ANOVA with Bonferroni *post hoc* testing). H and I, Western blot was carried out to assess STAT3‐Y703 and STAT3‐K685 expression levels. Quantitated data were shown as means ± SD (each group of mice, n = 9; ***P < .001, unpaired Student's *t* test; ^###^ *P* < .001, two‐way ANOVA with Bonferroni *post hoc* testing)

To assess the effects and mechanisms of WWP2 on AngII‐induced VSMCs proliferation and migration in vivo, that is the impact on hypertensive vascular disease, vascular smooth muscle WWP2 knockout (*SM22a Cre+; WWP2^FL/FL^*) mice and the control (*SM22a Cre‐; WWP2^FL/FL^*) mice were generated, and the mouse model of hypertensive angiopathy was established by AngII administration. Immunofluorescence (Figure 6A) and Western blot (Figure 6B) of aortic vascular tissue were used to detect the expression of WWP2 in *SM22a Cre+; WWP2^FL/FL^* and *SM22a Cre‐; WWP2^FL/FL^* mice, and efficient vascular smooth muscle‐specific silencing of WWP2 was demonstrated (Figure 6A‐C).

Arterial and venous thickening in mice was analysed by micro‐CT. The results showed that the degrees of arterial and venous thickening in *SM22a Cre+; WWP2^FL/FL^* mice were significantly decreased after stimulation by AngII compared with *SM22a Cre‐; WWP2^FL/FL^* mice (Figure 6D‐G). Moreover, venous thickening in AngII‐induced hypertension rescued in *SM22a Cre+; WWP2^FL/FL^* mice was more significant compared with arterial thickening in hypertension condition (Figure 6F,G).

In addition, whether WWP2 participates in VSMCs proliferation and migration by promoting STAT3‐K685 acetylation and STAT3‐Y705 phosphorylation in vivo was evaluated. To this end, STAT3‐K685 acetylation and STAT3‐Y705 phosphorylation levels were detected in AngII‐induced hypertensive angiopathy models in *SM22a Cre‐; WWP2^FL/FL^* and *SM22a Cre+; WWP2^FL/FL^* mice. The results showed that mice vascular smooth muscle‐specific WWP2 knockout significantly alleviated AngII‐induced STAT3‐K685 acetylation and STAT3‐Y705 phosphorylation (Figure 6H‐I), indicating that the molecular mechanisms of WWP2‐regulated STAT3‐K685 acetylation and STAT3‐Y705 phosphorylation were consistent in vivo and in vitro.

### 3.7 Mice vascular smooth muscle‐specific WWP2 knockout reduces hypertensive vascular thickening and systolic blood pressure

**FIGURE 7:**
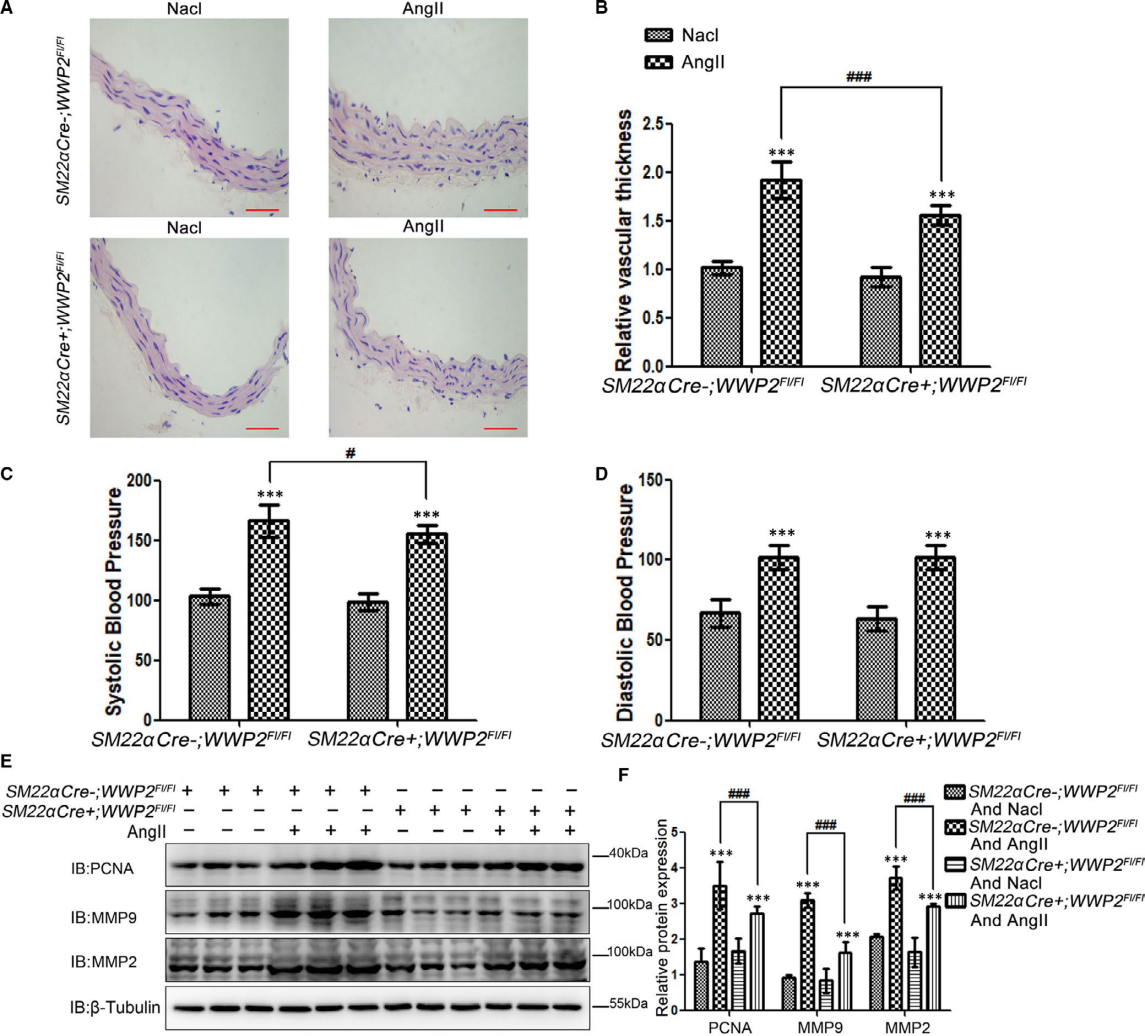
Mice vascular smooth muscle WWP2 knockout significantly relieves AngII‐induced hypertensive vascular thickening and systolic blood pressure. A and B, *SM22α Cre‐;WWP2^FL/FL^* and *SM22α Cre‐;WWP2^FL/FL^* mice induced by AngII or Nacl for 14 days. HE staining was carried out to assess aortic blood vessel thickness from *SM22α Cre‐;WWP2^FL/FL^* and *SM22α Cre‐;WWP2^FL/FL^* mice, and the aortic slices of each mouse were taken from the same position. Scale bar 100µm. Quantitated data were shown as means ± SD (each group of mice, n = 9; ***P < .001, unpaired Student's *t* test; ^###^ *P* < .001, two‐way ANOVA with Bonferroni *post hoc* testing). C and D, Systolic and diastolic blood pressures are measured, and quantitated data were shown as means ± SD (each group of mice, n = 9; ***P < .001, unpaired Student's *t* test; ^#^ *P* < .05, two‐way ANOVA with Bonferroni *post hoc* testing). E and F, Total protein was obtained from aortic vascular tissue of *SM22α Cre‐;WWP2^FL/FL^* and *SM22α Cre‐;WWP2^FL/FL^* mice, and Western blot was carried out to assess the expression levels of PCNA, MMP2 and MMP9. Quantitated data were shown as means ± SD (each group of mice, n = 9; ***P < .001, unpaired Student's *t* test; ^###^ *P* < .001, two‐way ANOVA with Bonferroni *post hoc* testing)

Next, our results showed that *SM22a Cre+; WWP2^FL/FL^* mice significantly relieved AngII‐induced hypertensive vascular thickening (Figure 7A‐B) compared with *SM22a Cre‐; WWP2^FL/FL^* mice, as well as significantly reduced AngII‐induced elevated systolic blood pressure (Figure 7C). Western blot showed that *SM22a Cre+; WWP2^FL/FL^* mice significantly decreased AngII‐induced proliferation and migration protein makers PCNA, MMP2 and MMP9 up‐regulation (Figure 7E‐F).

In summary, in vivo and in vitro assays confirmed that WWP2 formed a complex with SIRT1‐STAT3, inhibiting the interaction between SIRT1 and STAT3, then reducing the inhibitory effect of SIRT1 on STAT3, ensuing promoting STAT3‐K685 acetylation and STAT3‐Y705 phosphorylation, which finally promoted AngII‐induced proliferation, migration and phenotypic transformation in VSMCs and hypertensive angiopathy in mice. Therefore, targeted knockout of WWP2 in vascular smooth muscle cells may be a new tool for the prevention and treatment of hypertensive vascular diseases such as stroke, kidney and heart failure.

## 4 DISCUSSION

The following important new findings were obtained in this study: (a) the expression of WWP2 was significantly enhanced in AngII‐induced VSMCs proliferation, migration and phenotypic transformation models. (b) Vascular smooth muscle‐specific WWP2 knockout mice (*SM22a Cre+; WWP2^FL/FL^*) showed significantly alleviated in AngII‐induced hypertensive angiopathy. (c) In vitro experiments showed that overexpression of WWP2 significantly increased AngII‐induced VSMCs proliferation, migration and phenotypic transformation, whereas WWP2 knockdown had opposite effects. (d) Mechanistically, WWP2 is a novel interacting protein of SIRT1 and STAT3. Moreover, WWP2 formed a complex with SIRT1‐STAT3, inhibiting the interaction between SIRT1 and STAT3, then reducing the inhibitory effect of SIRT1 on STAT3, ensuing promoting STAT3‐K685 acetylation and STAT3‐Y705 phosphorylation. (e) Finally, these mechanisms were detected both in vivo and in vitro (Figure 3E,F and Figure 6H,I).

WWP2 is an E3 ubiquitin ligase of the HECT‐type NEDD4 family, which participates in the regulation of various physiological and pathological processes by interacting with different substrates.^16^, ^17^, ^18^, ^19^, ^20^, ^21^, ^22^ Initially, WWP2 was considered a tumour‐promoting factor involved in the development of tumours by regulating PETN and Smad.^26^, ^27^ Currently, WWP2 is considered to be involved in various life activities, such as development and ossification.^16^, ^19^, ^20^, ^21^, ^22^ In previous studies, WWP2 was discovered to be highly expressed in diabetic cardiomyopathic heart.^28^ In primary cardiac fibroblasts, TGFβ1 irritates the N‐terminal subtype of WWP2 to enter the nucleus, subsequently strengthening the activity of WWP2‐FL to promote the combining with Smad2, and may promote its monoubiquitination, thus activating the downstream Pro‐fibrogenic gene programme.^29^, ^30^ Therefore, WWP2 plays an important role in controlling pathological myocardial fibrosis and heart failure and improving the clinical prognosis of patients with heart disease. In our previous study, WWP2 interacts with the BRCT domain of PARP1 and ubiquitinates its K249 and K418. WWP2 cannot promote the ubiquitination levels of PARP1 in *MycCre + WWP2 ^FL/FL^* mice induced by isoprenaline (ISO) and the expression levels of PARP1 increases, thus promoting ISO triggered cardiac remodelling. Therefore, WWP2 can degrade PARP1 and protect it from ISO triggered cardiac remodelling. This provides a basis for the study of the treatment of heart remodelling‐related diseases.^31^ In addition, our another previous study showed that the silencing of endothelial/ myeloid‐specific WWP2 gene markedly increased the endothelial injury and vascular remodelling induced by Ang II. WWP2 promotes the degradation of lysine residue 174 (K174)‐Septin4 through ubiquitin proteasome, thus inhibiting the formation of Septin4‐PARP1 endothelial damage complex.^32^ Therefore, WWP2‐Septin4 pathway may be a new target for the treatment of atherosclerosis and hypertension.^33^ In this study, in vivo and in vitro experiments revealed key roles for WWP2 in VSMCs proliferation, migration and phenotypic transformation, as well as hypertensive angiopathy. Furthermore, it was found that the E3 ubiquitin ligase WWP2 played a new non‐ubiquitination function, *that is* competitive binding to SIRT1 and STAT3, antagonizing STAT3 deacetylation by SIRT1, ensuing promoting STAT3 acetylation, thus enhancing STAT3 phosphorylation. In conclusion, WWP2 has not been served as a therapeutic target; however, it is an important potential target for targeted treatment of various cardiovascular‐related diseases.

STAT3 is an important member of the family of kinase signal transduction and transcriptional activators. It participates in a series of cardiovascular diseases such as hypertension, atherosclerosis, myocardial infarction and ischaemia‐reperfusion injury by regulating biological events such as chronic inflammation, oxidative stress, cell proliferation and apoptosis.^34^, ^35^, ^36^, ^37^ STAT3 and IL‐6 double‐knockout mice significantly reduce AngII‐induced STAT3 phosphorylation, which counteracts the occurrence of AngII‐induced hypertensive angiopathy.^5^, ^6^ Additionally, the atherosclerotic area in the aortic root of oxidized phospholipid‐mediated STAT3^−/−^ mice decreases significantly compared with that of STAT3^+/+^ mice.^4^ Meanwhile, STAT3 knockout in VSMCs significantly reduces AngII‐induced VSMCs proliferation and migration.^38^, ^39^ Moreover, studies have demonstrated that STAT3 promotes VSMCs proliferation and migration mainly depends on the phosphorylation activity, and such phosphorylation activity is regulated by STAT3 acetylation.^15^, ^40^ In this study, it was found for the first time that STAT3 phosphorylation and acetylation were regulated by WWP2, with the regulatory loci including STAT3‐K685 and STAT3‐Y705, which further confirmed the roles of WWP2 in VSMCs proliferation and migration, as well as hypertensive angiopathy.

We found that SIRT1 was another new protein interacting with WWP2, by exploring the specific mechanism by which WWP2 regulates STAT3 acetylation and phosphorylation. SIRT1 is a member of the mammalian family of Sirtuins and a nicotinamide adenine dinucleotide (NAD^+^)‐dependent deacetylase.^10^, ^15^ Studies have shown that SIRT1 deacetylates STAT3, ensuing inhibiting STAT3 acetylation and phosphorylation.^14^, ^15^ In addition, SIRT1 plays an important protective role in the cardiovascular system through its functions on STAT3 deacetylation.^10^, ^11^, ^12^, ^13^, ^14^, ^15^ A study revealed that atherosclerosis in SIRT1^+/‐^Apo E^−/−^ mice is decreased significantly compared with ApoE^−/−^ counterparts.^10^ SIRT1 can also suppress AngII‐induced VSMCs proliferation and migration as well as phenotypic transformation, counteracting hypertensive angiopathy.^11^, ^12^, ^13^ In this study, WWP2 was identified as a new protein interacting with SIRT1. WWP2 formed a complex with SIRT1‐STAT3 to complete with the interaction between SIRT1 and STAT3, ensuing antagonizing STAT3 deacetylation by SIRT1. This resulted in enhanced STAT3‐K685 acetylation and STAT3‐Y705 phosphorylation, ensuing promoting VSMCs proliferation, migration and phenotypic transformation.

In this study, WWP2 was found to have important regulatory roles in AngII‐induced VSMCs proliferation, migration and phenotypic transformation in vitro (WWP2 knockdown/overexpression in VSMCs), as well as hypertensive angiopathy in vivo (vascular smooth muscle‐specific WWP2 knockout mice). Moreover, this study revealed that WWP2 promoted STAT3‐K685 acetylation and STAT3‐Y705 phosphorylation by antagonizing the modification effect of SIRT1 on STAT3. These findings suggested a new regulatory tool for counteracting AngII‐induced VSMCs proliferation, migration and phenotypic transformation as well as hypertensive angiopathy, providing novel insights into the treatment of hypertensive vascular diseases such as stroke, and heart and kidney failure. It is of great significance to further investigate whether other E3 ubiquitinated ligases are involved in hypertensive angiopathy. In addition, the roles of WWP2 in other cardiovascular diseases and whether WWP2 has functions in patients with hypertensive angiopathy should be further assessed.

In conclusion, WWP2 formed a complex with SIRT1‐STAT3, inhibiting the interaction between SIRT1 and STAT3, then reducing the inhibitory effect of SIRT1 on STAT3, ensuing enhancing STAT3‐K685 acetylation and STAT3‐Y705 phosphorylation, which finally promoted AngII‐induced proliferation, migration and phenotypic transformation in VSMCs and hypertensive angiopathy in mice. In vivo and in vitro vascular smooth muscle‐specific WWP2 knockout alleviates hypertensive angiopathy. This study provides new insights into the treatment of hypertensive vascular diseases such as stroke, and heart and kidney failure.

## CONFLICT OF INTEREST

The authors confirm that there are no conflicts of interest.

## AUTHOR CONTRIBUTIONS

All authors have read and approved the content and agree with publication in this journal. YS and LC guided this study. NZ and YZ designed and conducted the transgenic mice and mechanism part of the experiments. SY YT and SL contributed in plasmids construct and Western blots. NZ and YZ wrote the paper.

## Supporting information

Supplementary Material

## Data Availability

The data used to support the findings of this study are included within the article.
