# Supplementary material for: WWP2 regulates SIRT1‐STAT3 acetylation and phosphorylation involved in hypertensive angiopathy
Source: J Cell Mol Med. 2020 Jul 5;24(16):9041–54. doi: 10.1111/jcmm.15538 (PMC7417706; doi:10.1111/jcmm.15538)
Supplement: Supplementary file 1 — Supplementary Material [file JCMM-24-9041-s001.docx]

**ONLINE SUPPLEMENT**

**WWP2 regulates SIRT1-STAT3 acetylation and phosphorylation involved in hypertensive angiopathy.**

Authors: Ying Zhang^1#^, Shilong You^1^, Yichen Tian^1^, Saien Lu^1^, Liu Cao^2^*, Yingxian Sun^1^*, Naijin Zhang^1^*

**# first author:** Ying Zhang

*** Corresponding author:** Naijin Zhang; Yingxian Sun; Liu Cao

^1^ Department of Cardiology, the First Hospital of China Medical University, Shenyang, Liaoning, China;

^2^ Key Laboratory of Medical Cell Biology, Ministry of Education; Institute of Translational Medicine, China Medical University; Liaoning Province Collaborative Innovation Center of Aging Related Disease Diagnosis and Treatment and Prevention, Shenyang, Liaoning, China.

**Catalogue:**

1: Supplementary figure 1

2: Supplementary figure 2

3: Supplementary figure 3

**
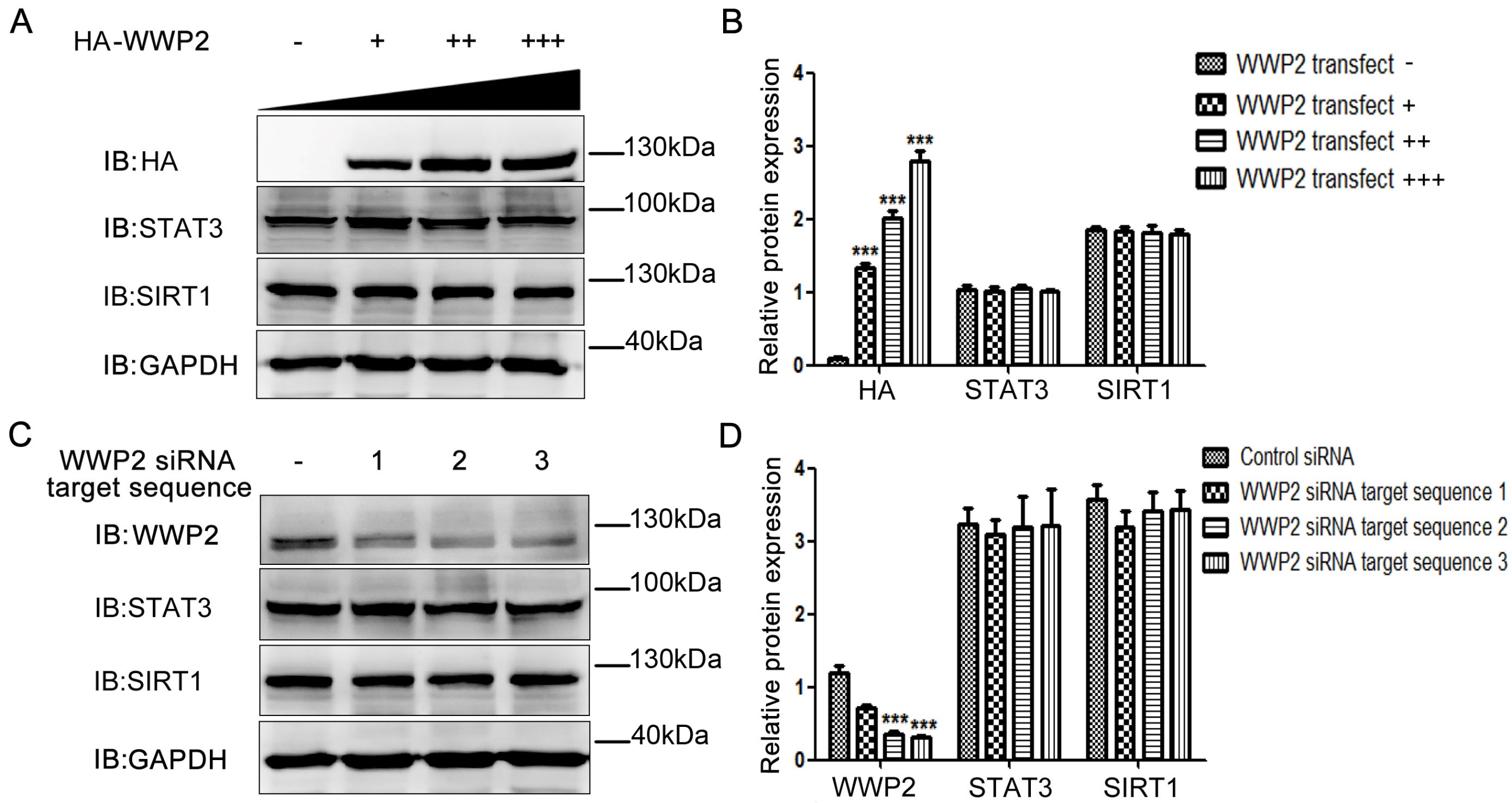
**

**Supplement Figure 1 WWP2 is not involved in proteasome dependent STAT3 and SIRT1 degradation.**

(A) Western blot was carried out to assess STAT3 and SIRT1 expression levels with overexpression of HA-WWP2 gradually. (B) Quantification of Western blot data was shown as means ± SD (***P<0.001, unpaired Student’s *t*-test). (C) Three target sequences of siRNA-WWP2 were transfected, and the efficiency of WWP2 knockdown, STAT3 and SIRT1 expression were determined. (D) Quantification of Western blot data was shown as means ± SD (***P<0.001, unpaired Student’s *t*-test).


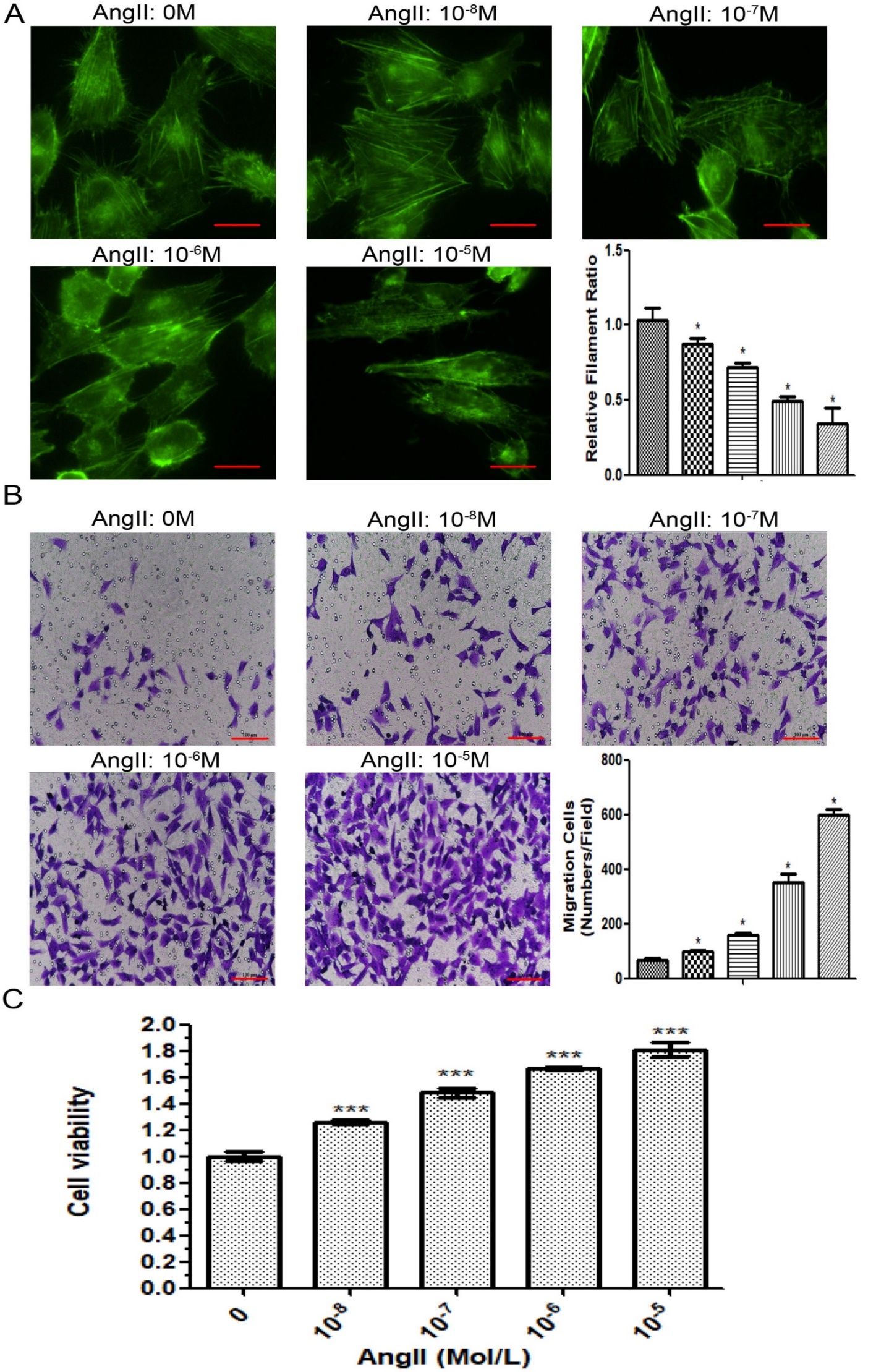


**Supplement Figure 2 Angiotensin II induced HAVSMCs proliferation, migration and phenotypic transformation.**

(A) Phalloidine dye was measured to assess the HAVSMCs phenotypic transformation with gradient of angiotensin II and intracellular myofilaments were labeled with green fluorescence. Quantitated data were shown as means ± SD (P<0.001). (B) Transwell was measured to assess the HAVSMCs migration with gradient of angiotensin II and quantitated data were shown as means ± SD (P<0.001). (C) CCK8 was measured to assess HAVSMCs proliferation with gradient of angiotensin II and quantitated data were shown as means ± SD (P<0.001).


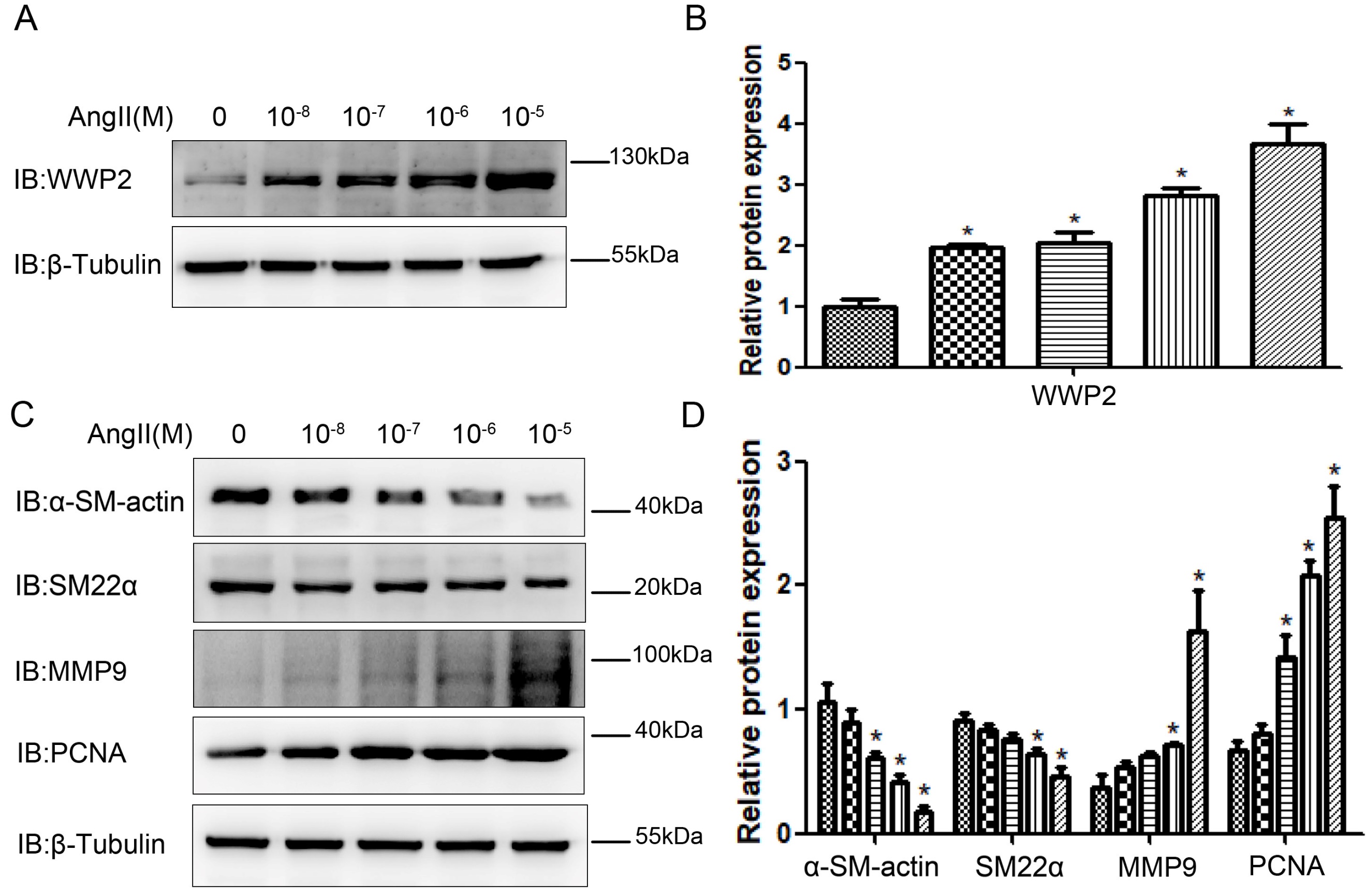


**Supplement Figure 3 WWP2 increases gradually with angiotensin II-induced HAVSMCs proliferation, migration and phenotypic transformation.**

(A) WWP2 were examined by Western blot with a concentration gradient of angiotensin II and (B) quantification of results as means ± SD (P<0.001). (C) PCNA, MMP2, MMP9, α-SM-actin and SM22α levels were examined by Western blot with a concentration gradient of angiotensin II and (D) quantification of results as means ± SD (P<0.001).
